# Ultrasound of Primary Aneurysmal Bone Cyst

**DOI:** 10.1155/2014/101069

**Published:** 2014-01-23

**Authors:** Katrina N. Glazebrook, Gary L. Keeney, Michael G. Rock

**Affiliations:** Mayo Clinic, 200 First Street SW, Rochester, MN 55905, USA

## Abstract

Aneurysmal bone cysts (ABC) are rare, benign, expansile lesions of bone often found in the metaphyses of long bones in pediatric and young adult population. Multiple fluid levels are typically seen on imaging with magnetic resonance imaging (MRI) or computed tomography (CT). We describe a case of a primary ABC in the fibula of a 34-year-old man diagnosed on ultrasound with a mobile fluid level demonstrated sonographically.

## 1. Introduction

Aneurysmal bone cysts (ABC) are rare, benign, expansile lesions of bone most commonly found in the metaphyses of long bones in pediatric and young adult population. The lesion is characterized by blood filled spaces separated by fibrous septa that may contain osteoclast-like giant cells. It can be a primary lesion or arise adjacent to other benign or malignant osseous processes. Imaging with magnetic resonance imaging (MRI) or computed tomography (CT) typically shows multiple fluid levels. Bone lesions are not typically evaluated with ultrasound as the sound waves are not able to penetrate the cortex. If however the cortex is thinned, expanded, or disrupted, ultrasound can identify primary and secondary bone tumors. Ultrasound is often used as a first imaging study for evaluation of palpable superficial masses. Recognition of a lesion to be originating from the bone rather than soft tissue and identification of mobile fluid/fluid levels can suggest the diagnosis of aneurysmal bone cyst and direct the patient to the appropriate treatment. Little has been written in the literature about the sonographic appearance of ABC. We describe the ultrasound, radiographic, and MRI appearance of an aneurysmal bone cyst in the distal fibula.

## 2. Case Report

A 34-year-old man presented to his family practitioner with a two-month history of swelling and discomfort in the left lateral lower leg just above his ankle. There was no preceding history of trauma. Physical examination revealed soft tissue fullness at the junction of the proximal two-thirds and distal one-third of the left fibula which was painful to touch. The patient was sent for an ultrasound for evaluation and possibly to aspirate a presumed ganglion cyst.

**Figure 1:**
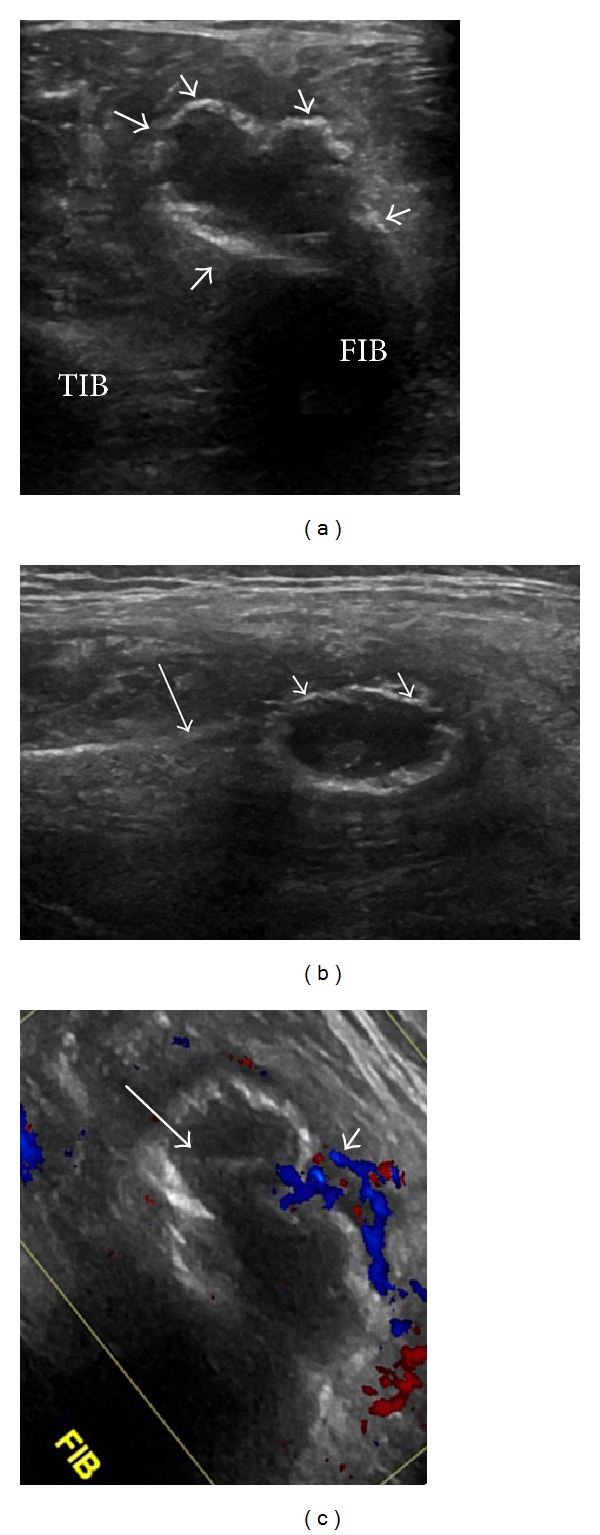
Ultrasound of the distal left fibula in a 34-year-old man. ((a) and (b)) Transverse and longitudinal US of the distal left fibula demonstrates an expansile, cortically centered bone lesion extending from the fibular metadiaphysis into the anterior compartment of the calf with a thin echogenic rim of cortex (short arrows) which was contiguous with the underlying fibular cortex (long arrow). TIB: tibia, FIB: fibula. (c) Transverse scan of the distal fibula has been rotated to the same orientation as the MRI (see Figure 2). This shows increased vascularity in the adjacent soft tissue about the fibular lesion on color Doppler evaluation (short arrow), without vascularity seen within the lesion. Fluid level is noted (long arrow).

Ultrasound was performed using a General Electric Healthcare Logiq E9 linear ML 6–15 MHz transducer (GE Healthcare Wauwatosa, WI). A cortically based lesion was noted arising from the anterolateral cortex of the fibula with elevation of the periosteum and a thin rim of echogenicity surrounding the mass, presumed to be a thin shell of bone (Figure 1) which appeared intact without adjacent soft tissue mass. Two fluid-fluid levels were noted within the mass which were mobile on patient rotation indicating the cystic nature of the lesion's contents. No internal soft tissue mass extending from the fibular medullary canal was noted. There was increased vascularity in the adjacent soft tissues on color Doppler evaluation consistent with inflammatory changes. The most likely diagnosis was a cortically based aneurysmal bone cyst (ABC) and not a soft tissue solid or cystic mass. In view of the periosteal elevation or sonographic “Codman's triangle,” the mass was thought to be centered and to have originated within the bone rather than to be a soft tissue mass such as a ganglion cyst which had eroded into the bone. A Brodie abscess or subacute osteomyelitis could have this appearance as symptoms can be indolent, but these tend to be metaphyseal and centrally located. The adjacent fibular cortex was normal with no adjacent soft tissue mass to suggest an underlying aggressive bone lesion such as osteosarcoma or metastasis with secondary ABC formation.

**Figure 2:**
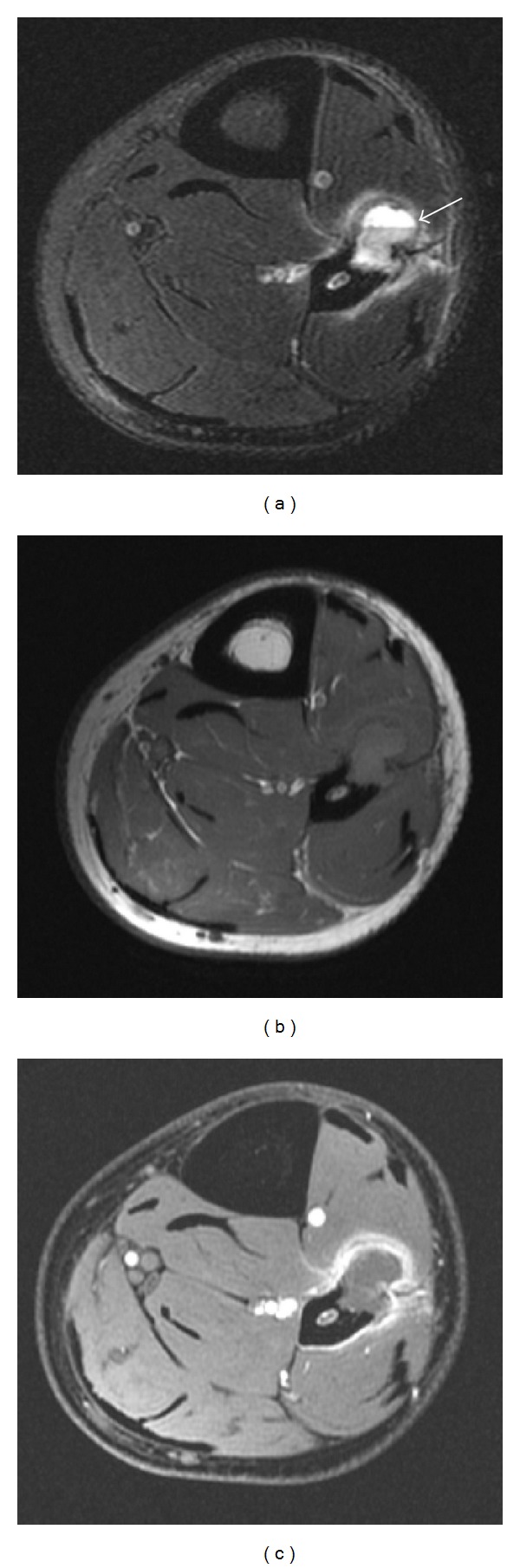
MRI of the distal fibula. (a) Axial T2-weighted MRI with fat saturation demonstrates a cortically based lesion within the distal fibula with a large fluid-fluid level (arrow). Peripheral increased T2 signal around the ABC is consistent with inflammatory changes. (b) Axial T1-weighted MRI shows the lesion is cortically based and has intermediate T1 signal within the mass. (c) Axial spoiled gradient with fat saturation following gadolinium shows peripheral enhancement only corresponding to the increased vascularity seen on color Doppler evaluation (Figure 1(c)).

MRI was obtained for preoperative planning, consisting of axial and sagittal T1-weighted and fat saturated T2-weighted images with axial and sagittal fat saturated spoiled gradient echo sequence after gadolinium administration. This confirmed a cortically based mass arising from the fibula extending into the anterior compartment, with fluid-fluid levels seen on the T2-weighted sequence (Figure 2). There was peripheral enhancement and increased T2 signal in the adjacent soft tissues and fibular marrow consistent with inflammatory changes corresponding to the increased vascularity seen sonographically. No primary lesion could be seen and this was presumed to be a primary cortical ABC.

**Figure 3:**
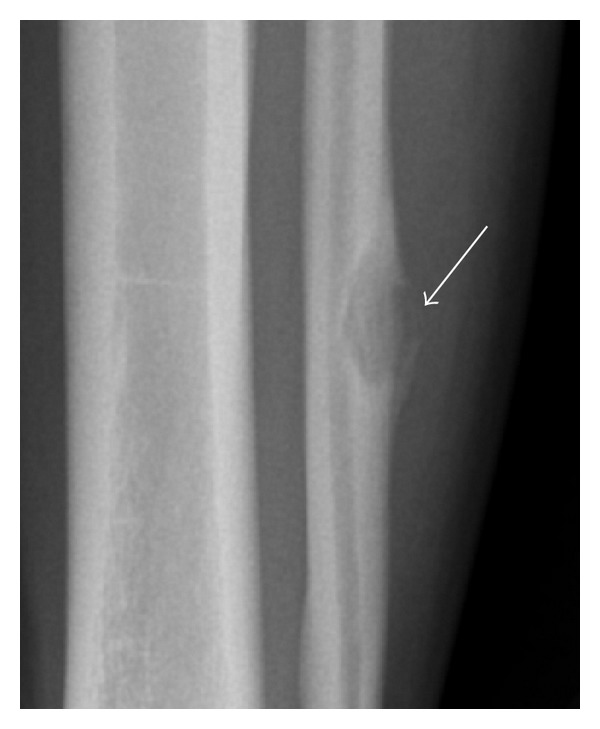
AP radiograph of the distal left fibula shows a lytic lesion with narrow zone of transition within the fibular cortex. There is periosteal elevation and a thin rim of bone surrounding the lesion (arrow).

**Figure 4:**
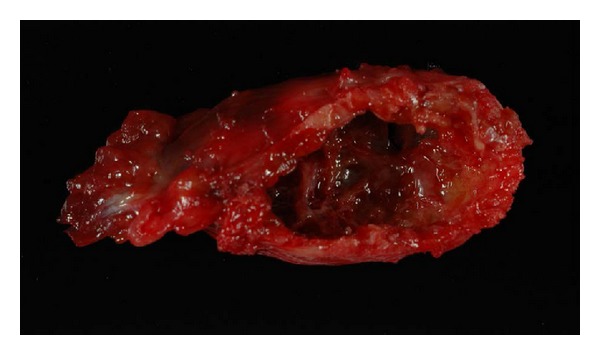
Photograph of the gross specimen shows the cystic space within the cortex of the bone.

Radiograph demonstrated a cortically based lesion with a narrow zone of transition, elevating the periosteum with a thin shell of bone (Figure 3). No other lesions were seen in the fibula or tibia. The patient underwent surgical excision of the lesion with curettage. A histologic diagnosis of aneurysmal bone cyst was made forming a 3.2 × 1.7 × 0.9 cm cystic mass (Figure 4). Microscopically, a cystic space with peripheral shell of reactive bone with numerous osteoclast giant cells and scattered histiocytes and lymphocytes was noted. Some of the histiocytes contain hemosiderin.

## 3. Discussion

Aneurysmal bone cyst is a nonneoplastic expansile lesion of bone, mainly affecting children and young adults [1]. In a study of 238 cases of ABC by Vergel De Dios et al., for patients with ABC of the long bones, 86% of patients were younger than 20 years (range of 1.5 to 69 years) [2]. More than 80% of lesions were in the long bones, commonly in the metaphysis, flat bones, or spinal column. Pathologically the lesion consists of channels or spaces separated by fibrous septa which may contain osteoclast-like giant cells and bone trabeculae. Thick new bone formation at the edge of the lesions was present in 52% in the long bones with calcified matrix; a cartilage aura and adjacent myxoid regions were seen in 11 to 16% of long bone ABC. Multiple bones may be affected. There is a rare solid variant which does not contain the cavernous spaces but otherwise has identical histologic findings. Pain and swelling are the common clinical presentations as in our case. Radiographically, ABCs were found centrally or eccentrically in the medulla in 23% and 58%, respectively. In 19% of cases the ABCs were centered in the cortex or on the surface of bone as in our case. ABCs involved the metaphysis or metadiaphysis in 58% of cases. Periosteal new bone was seen in 66% of cases with a thin rim of bone over the external surface of the ABC in 63% of cases which can be a helpful radiographic sign. It can exist as a primary lesion or be secondary to either a benign bone lesion such as chondroblastoma or a malignant bone tumor. In our case no additional bone lesion was seen on imaging or histologically.

High resolution ultrasound is increasingly used for initial assessment of ambiguous musculoskeletal soft tissue lesions and for sonographically guided biopsy [3]. Ultrasound is generally not helpful in intramedullary bone lesions as sound waves cannot penetrate the normal cortex [4]. Ultrasound can, however, readily identify primary and secondary bone tumors where there are cortical disruption and soft tissue masses [4]. These areas may then be targeted for percutaneous biopsy under ultrasound guidance which is a quick procedure without ionizing radiation. Ultrasound may also be useful in evaluation of postoperative sites for tumor recurrence particularly if there is significant artefact on MRI or CT due to orthopedic hardware. If the cortex is sufficiently thinned, the sound waves may have sufficient sound transmission to identify the underlying bone lesion. Fornage et al. showed the cystic nature of a calcaneal lesion with ultrasound and used the US to guide a needle aspiration for confirmation of a calcaneal cyst [5]. Haber et al. described the ultrasound findings in a primary ABC in the scapula in a 1-year-old seen on radiograph as an expansile lesion [6]. They found a cystic mass with a thin echogenic shell and multiple intraosseous fluid levels. Suh and Han described fluid levels in large expansile lesion in the ilium [7] with CT for confirmation. In both cases, the cortices of the affected bones were markedly thinned allowing sound to be transmitted and for characterization of the internal structure of the lesions. In both cases, the fluid levels were seen to move with changes in position of the patients as in our case, confirming the cystic nature of the lesion.

Fluid levels seen with ABC have been well documented with CT and MRI [8, 9]. Since the initial description, fluid-fluid levels have been described in many bone pathologies and so this finding has become a nonspecific observation. O'Donnell and Saifuddin evaluated the prevalence and diagnostic significance of fluid-fluid levels (FFLs) in focal bone lesions in 738 consecutive patients [10]. FFLs were present in 83 patients (11.2%). Malignant neoplasms most commonly showed FFLs in less than third of the lesion. With increase in the total volume of FFLs, there was a decrease in percentage of malignancy. There were no malignant lesions if 100% of the lesion showed FFL changes. Some aggressive high grade predominantly necrotic bone tumors, particularly telangiectatic osteosarcomas, may have greater than 2/3 of the lesion containing FFLs. These tumors often show a small solid component but differentiation with ABC's can be difficult on MRI. Radiographs may be helpful; however, ABCs can show an aggressive appearance and malignancies may have a more indolent radiographic appearance. Sundaram et al. describe four cases of osteosarcoma with clinical and imaging findings suggestive of simple or aneurysmal bone cyst radiographically [11]. One tumor was a giant cell-rich variant of osteosarcoma with focal aneurysmal bone cyst-like areas within the navicular bone. MRI showed fluid-fluid levels with expansion of the bone and no soft tissue mass. Clinical features and radiographs should be used to differentiate between TOS and ABC. Microscopically, the tumors were not cystic or telangiectatic but were conventional osteosarcoma and osteoclast-rich osteosarcoma and so did not pose a pathologic dilemma.

Treatment of ABC includes curettage with bone grafting if technically possible. Curettage without bone grafting can be safely performed with protected weightbearing until healing has occurred [12]. Radiation had been employed in the past, though this is now avoided to prevent post radiation sarcomas. Recurrence can occur in up to 19% of cases.

## 4. Conclusion

Ultrasound may be the first imaging test to evaluate a superficial mass. It is important for the radiologist to be able to recognize a bone rather than soft tissue neoplasm. If the cortex is sufficiently thinned, then ultrasound can demonstrate fluid levels suggesting an aneurysmal bone cyst. Appropriate additional imaging for preoperative planning and surgical management can then be performed without delay in diagnosis.
